# Supplementary material for: Modeling glioblastoma heterogeneity as a dynamic network of cell states
Source: Mol Syst Biol. 2021 Sep 16;17(9):e10105. doi: 10.15252/msb.202010105 (PMC8444284; doi:10.15252/msb.202010105)
Supplement: Supplementary file 5 — Source Data for Figure 3 [file MSB-17-e10105-s001.zip › Figure3A_sourcedata/GSEA_3065/nosigdb_state4.GseaPreranked.1622718430994/RICHARDS_NOWAKOWSKI_SCIENCE_2017_ASTROCYTE_UPREG.html]

Details for gene set RICHARDS\_NOWAKOWSKI\_SCIENCE\_2017\_ASTROCYTE\_UPREG[GSEA]

|  || Dataset | state4 |
| Phenotype | NoPhenotypeAvailable |
| Upregulated in class | na\_pos |
| GeneSet | RICHARDS\_NOWAKOWSKI\_SCIENCE\_2017\_ASTROCYTE\_UPREG |
| Enrichment Score (ES) | 0.35981375 |
| Normalized Enrichment Score (NES) | 1.7989726 |
| Nominal p-value | 0.0 |
| FDR q-value | 0.008920638 |
| FWER p-Value | 0.078 |
Table: GSEA Results Summary

  

Fig 1: Enrichment plot: RICHARDS\_NOWAKOWSKI\_SCIENCE\_2017\_ASTROCYTE\_UPREG      
 Profile of the Running ES Score & Positions of GeneSet Members on the Rank Ordered List

  

| PROBE | GENE SYMBOL | GENE\_TITLE | RANK IN GENE LIST | RANK METRIC SCORE | RUNNING ES | CORE ENRICHMENT || 1 | GFAP |  |  | 0 | 0.995 | 0.0210 | Yes |
| 2 | PMP2 |  |  | 3 | 0.727 | 0.0360 | Yes |
| 3 | SAT1 |  |  | 4 | 0.669 | 0.0501 | Yes |
| 4 | FABP7 |  |  | 5 | 0.656 | 0.0639 | Yes |
| 5 | S100B |  |  | 6 | 0.630 | 0.0772 | Yes |
| 6 | SPARCL1 |  |  | 17 | 0.416 | 0.0842 | Yes |
| 7 | OLFM2 |  |  | 20 | 0.396 | 0.0923 | Yes |
| 8 | RAB31 |  |  | 21 | 0.388 | 0.1004 | Yes |
| 9 | TOMM7 |  |  | 22 | 0.380 | 0.1084 | Yes |
| 10 | FABP5 |  |  | 23 | 0.375 | 0.1164 | Yes |
| 11 | HOPX |  |  | 28 | 0.360 | 0.1233 | Yes |
| 12 | QKI |  |  | 31 | 0.345 | 0.1302 | Yes |
| 13 | ZMAT3 |  |  | 34 | 0.338 | 0.1370 | Yes |
| 14 | MT3 |  |  | 38 | 0.318 | 0.1431 | Yes |
| 15 | HEY1 |  |  | 39 | 0.318 | 0.1498 | Yes |
| 16 | NEAT1 |  |  | 41 | 0.315 | 0.1563 | Yes |
| 17 | SOX9 |  |  | 48 | 0.299 | 0.1616 | Yes |
| 18 | SNX3 |  |  | 51 | 0.297 | 0.1675 | Yes |
| 19 | ID2 |  |  | 56 | 0.292 | 0.1730 | Yes |
| 20 | SEPT7 |  |  | 57 | 0.292 | 0.1791 | Yes |
| 21 | FHL1 |  |  | 62 | 0.286 | 0.1845 | Yes |
| 22 | SESN3 |  |  | 68 | 0.278 | 0.1895 | Yes |
| 23 | SCP2 |  |  | 83 | 0.261 | 0.1926 | Yes |
| 24 | ID1 |  |  | 84 | 0.261 | 0.1981 | Yes |
| 25 | FYN |  |  | 87 | 0.260 | 0.2032 | Yes |
| 26 | TXNIP |  |  | 92 | 0.254 | 0.2079 | Yes |
| 27 | CLU |  |  | 95 | 0.252 | 0.2129 | Yes |
| 28 | TTYH1 |  |  | 100 | 0.249 | 0.2174 | Yes |
| 29 | APCDD1 |  |  | 109 | 0.244 | 0.2212 | Yes |
| 30 | PEA15 |  |  | 112 | 0.243 | 0.2260 | Yes |
| 31 | FAM181B |  |  | 130 | 0.232 | 0.2280 | Yes |
| 32 | ZFP36L2 |  |  | 135 | 0.228 | 0.2321 | Yes |
| 33 | ENHO |  |  | 136 | 0.228 | 0.2369 | Yes |
| 34 | CXXC5 |  |  | 152 | 0.221 | 0.2390 | Yes |
| 35 | CHPT1 |  |  | 155 | 0.220 | 0.2432 | Yes |
| 36 | GATM |  |  | 156 | 0.219 | 0.2479 | Yes |
| 37 | ATP1B2 |  |  | 159 | 0.217 | 0.2521 | Yes |
| 38 | CNN3 |  |  | 160 | 0.217 | 0.2567 | Yes |
| 39 | CTNND2 |  |  | 164 | 0.215 | 0.2607 | Yes |
| 40 | RHOC |  |  | 169 | 0.214 | 0.2645 | Yes |
| 41 | TRIM47 |  |  | 175 | 0.212 | 0.2681 | Yes |
| 42 | LRRN3 |  |  | 181 | 0.211 | 0.2717 | Yes |
| 43 | POLR2L |  |  | 184 | 0.210 | 0.2758 | Yes |
| 44 | ZEB1 |  |  | 214 | 0.198 | 0.2750 | Yes |
| 45 | ITPR2 |  |  | 222 | 0.193 | 0.2779 | Yes |
| 46 | GNG12 |  |  | 231 | 0.190 | 0.2805 | Yes |
| 47 | TAOK3 |  |  | 234 | 0.190 | 0.2842 | Yes |
| 48 | BBX |  |  | 242 | 0.187 | 0.2869 | Yes |
| 49 | DCLK2 |  |  | 250 | 0.186 | 0.2896 | Yes |
| 50 | LYPD1 |  |  | 264 | 0.183 | 0.2913 | Yes |
| 51 | EDNRB |  |  | 271 | 0.180 | 0.2940 | Yes |
| 52 | ZBTB20 |  |  | 275 | 0.180 | 0.2973 | Yes |
| 53 | UBL3 |  |  | 278 | 0.179 | 0.3007 | Yes |
| 54 | ARHGEF6 |  |  | 284 | 0.178 | 0.3036 | Yes |
| 55 | NEK6 |  |  | 293 | 0.174 | 0.3060 | Yes |
| 56 | LITAF |  |  | 295 | 0.174 | 0.3095 | Yes |
| 57 | TRIM9 |  |  | 309 | 0.172 | 0.3109 | Yes |
| 58 | SEPT2 |  |  | 337 | 0.168 | 0.3098 | Yes |
| 59 | MT1X |  |  | 341 | 0.167 | 0.3128 | Yes |
| 60 | GPM6B |  |  | 346 | 0.166 | 0.3156 | Yes |
| 61 | KAT2B |  |  | 347 | 0.166 | 0.3191 | Yes |
| 62 | NDRG4 |  |  | 384 | 0.157 | 0.3162 | Yes |
| 63 | VEGFB |  |  | 414 | 0.149 | 0.3144 | Yes |
| 64 | PRDX1 |  |  | 416 | 0.149 | 0.3174 | Yes |
| 65 | PIK3R1 |  |  | 418 | 0.149 | 0.3203 | Yes |
| 66 | MRFAP1 |  |  | 424 | 0.148 | 0.3226 | Yes |
| 67 | ABAT |  |  | 435 | 0.145 | 0.3240 | Yes |
| 68 | SEMA6D |  |  | 454 | 0.143 | 0.3239 | Yes |
| 69 | SRI |  |  | 458 | 0.143 | 0.3264 | Yes |
| 70 | ZCCHC24 |  |  | 478 | 0.140 | 0.3261 | Yes |
| 71 | SRPK2 |  |  | 480 | 0.140 | 0.3289 | Yes |
| 72 | NACC2 |  |  | 485 | 0.139 | 0.3311 | Yes |
| 73 | RHOA |  |  | 492 | 0.139 | 0.3330 | Yes |
| 74 | ZFP36L1 |  |  | 497 | 0.137 | 0.3352 | Yes |
| 75 | HADHB |  |  | 503 | 0.136 | 0.3372 | Yes |
| 76 | LAMB2 |  |  | 510 | 0.135 | 0.3390 | Yes |
| 77 | TGFB2 |  |  | 537 | 0.132 | 0.3374 | Yes |
| 78 | RASSF2 |  |  | 564 | 0.129 | 0.3356 | Yes |
| 79 | MLC1 |  |  | 594 | 0.126 | 0.3333 | Yes |
| 80 | PSAP |  |  | 596 | 0.126 | 0.3358 | Yes |
| 81 | WSCD1 |  |  | 605 | 0.125 | 0.3371 | Yes |
| 82 | NDUFB5 |  |  | 614 | 0.124 | 0.3383 | Yes |
| 83 | SPATS2L |  |  | 620 | 0.123 | 0.3400 | Yes |
| 84 | PTPN13 |  |  | 631 | 0.122 | 0.3409 | Yes |
| 85 | TRAPPC3 |  |  | 669 | 0.118 | 0.3370 | Yes |
| 86 | MYO10 |  |  | 673 | 0.118 | 0.3390 | Yes |
| 87 | BDH2 |  |  | 688 | 0.116 | 0.3391 | Yes |
| 88 | HEPACAM |  |  | 703 | 0.113 | 0.3391 | Yes |
| 89 | GNG7 |  |  | 718 | 0.112 | 0.3390 | Yes |
| 90 | NTRK2 |  |  | 722 | 0.111 | 0.3408 | Yes |
| 91 | PHLPP1 |  |  | 740 | 0.109 | 0.3402 | Yes |
| 92 | PMP22 |  |  | 765 | 0.107 | 0.3384 | Yes |
| 93 | NADK2 |  |  | 766 | 0.106 | 0.3406 | Yes |
| 94 | CST3 |  |  | 792 | 0.104 | 0.3385 | Yes |
| 95 | C1orf61 |  |  | 803 | 0.104 | 0.3390 | Yes |
| 96 | ANXA5 |  |  | 814 | 0.103 | 0.3394 | Yes |
| 97 | BAALC |  |  | 816 | 0.103 | 0.3414 | Yes |
| 98 | GLUL |  |  | 819 | 0.102 | 0.3432 | Yes |
| 99 | FOS |  |  | 832 | 0.101 | 0.3433 | Yes |
| 100 | TRPS1 |  |  | 858 | 0.099 | 0.3411 | Yes |
| 101 | ADI1 |  |  | 863 | 0.099 | 0.3425 | Yes |
| 102 | NDUFB3 |  |  | 883 | 0.097 | 0.3413 | Yes |
| 103 | IGFBP5 |  |  | 901 | 0.096 | 0.3404 | Yes |
| 104 | COL11A1 |  |  | 905 | 0.095 | 0.3419 | Yes |
| 105 | HIPK2 |  |  | 909 | 0.095 | 0.3434 | Yes |
| 106 | IMPACT |  |  | 921 | 0.094 | 0.3435 | Yes |
| 107 | RNF13 |  |  | 942 | 0.092 | 0.3420 | Yes |
| 108 | EFCAB14 |  |  | 965 | 0.091 | 0.3401 | Yes |
| 109 | SOX2 |  |  | 975 | 0.090 | 0.3405 | Yes |
| 110 | NDUFA4 |  |  | 977 | 0.090 | 0.3422 | Yes |
| 111 | CLNS1A |  |  | 984 | 0.089 | 0.3431 | Yes |
| 112 | HEATR5A |  |  | 988 | 0.089 | 0.3444 | Yes |
| 113 | RGMA |  |  | 995 | 0.089 | 0.3452 | Yes |
| 114 | FAM19A5 |  |  | 1005 | 0.088 | 0.3456 | Yes |
| 115 | HTRA1 |  |  | 1008 | 0.088 | 0.3471 | Yes |
| 116 | SSPN |  |  | 1013 | 0.087 | 0.3482 | Yes |
| 117 | MAPK1 |  |  | 1014 | 0.087 | 0.3501 | Yes |
| 118 | ZIC1 |  |  | 1018 | 0.087 | 0.3514 | Yes |
| 119 | APC |  |  | 1029 | 0.086 | 0.3515 | Yes |
| 120 | CSPG5 |  |  | 1035 | 0.086 | 0.3524 | Yes |
| 121 | SEMA5A |  |  | 1036 | 0.085 | 0.3542 | Yes |
| 122 | CA2 |  |  | 1037 | 0.085 | 0.3560 | Yes |
| 123 | NOTCH3 |  |  | 1061 | 0.084 | 0.3539 | Yes |
| 124 | DTNA |  |  | 1063 | 0.084 | 0.3554 | Yes |
| 125 | SLC1A3 |  |  | 1069 | 0.083 | 0.3563 | Yes |
| 126 | SEMA6A |  |  | 1073 | 0.083 | 0.3576 | Yes |
| 127 | RASSF4 |  |  | 1099 | 0.082 | 0.3550 | Yes |
| 128 | AGT |  |  | 1103 | 0.082 | 0.3562 | Yes |
| 129 | ADD3 |  |  | 1118 | 0.081 | 0.3555 | Yes |
| 130 | AIF1L |  |  | 1119 | 0.081 | 0.3572 | Yes |
| 131 | ATF7 |  |  | 1141 | 0.079 | 0.3553 | Yes |
| 132 | DDAH1 |  |  | 1153 | 0.078 | 0.3551 | Yes |
| 133 | RFTN2 |  |  | 1157 | 0.078 | 0.3562 | Yes |
| 134 | SLC4A4 |  |  | 1160 | 0.078 | 0.3575 | Yes |
| 135 | HIST2H2BE |  |  | 1165 | 0.078 | 0.3585 | Yes |
| 136 | ALDH7A1 |  |  | 1172 | 0.078 | 0.3591 | Yes |
| 137 | RHOBTB3 |  |  | 1184 | 0.077 | 0.3588 | Yes |
| 138 | TIMP2 |  |  | 1194 | 0.076 | 0.3589 | Yes |
| 139 | ETV1 |  |  | 1199 | 0.076 | 0.3598 | Yes |
| 140 | TFPI |  |  | 1213 | 0.075 | 0.3592 | No |
| 141 | PLEKHG1 |  |  | 1250 | 0.073 | 0.3545 | No |
| 142 | DOCK1 |  |  | 1252 | 0.073 | 0.3559 | No |
| 143 | ARHGAP5 |  |  | 1259 | 0.073 | 0.3564 | No |
| 144 | ADCY2 |  |  | 1264 | 0.073 | 0.3573 | No |
| 145 | RAB13 |  |  | 1270 | 0.072 | 0.3579 | No |
| 146 | SCD5 |  |  | 1290 | 0.071 | 0.3562 | No |
| 147 | GLUD1 |  |  | 1295 | 0.071 | 0.3570 | No |
| 148 | CHP1 |  |  | 1347 | 0.069 | 0.3497 | No |
| 149 | VIM |  |  | 1350 | 0.069 | 0.3508 | No |
| 150 | SLC25A18 |  |  | 1363 | 0.068 | 0.3502 | No |
| 151 | OPHN1 |  |  | 1390 | 0.067 | 0.3471 | No |
| 152 | KANK1 |  |  | 1395 | 0.067 | 0.3478 | No |
| 153 | FERMT2 |  |  | 1426 | 0.065 | 0.3441 | No |
| 154 | HES1 |  |  | 1442 | 0.065 | 0.3429 | No |
| 155 | DNASE2 |  |  | 1462 | 0.063 | 0.3409 | No |
| 156 | NPAS3 |  |  | 1483 | 0.062 | 0.3388 | No |
| 157 | RHBDD2 |  |  | 1484 | 0.062 | 0.3401 | No |
| 158 | CASC4 |  |  | 1487 | 0.062 | 0.3411 | No |
| 159 | TRIB2 |  |  | 1496 | 0.062 | 0.3410 | No |
| 160 | LRP4 |  |  | 1502 | 0.062 | 0.3415 | No |
| 161 | LHX2 |  |  | 1534 | 0.061 | 0.3374 | No |
| 162 | ENKUR |  |  | 1537 | 0.061 | 0.3384 | No |
| 163 | PBXIP1 |  |  | 1539 | 0.060 | 0.3395 | No |
| 164 | MT2A |  |  | 1542 | 0.060 | 0.3404 | No |
| 165 | PEX2 |  |  | 1546 | 0.060 | 0.3412 | No |
| 166 | LAP3 |  |  | 1570 | 0.059 | 0.3385 | No |
| 167 | TSPAN3 |  |  | 1584 | 0.059 | 0.3375 | No |
| 168 | ZC3HAV1 |  |  | 1592 | 0.059 | 0.3375 | No |
| 169 | SAMD8 |  |  | 1596 | 0.059 | 0.3382 | No |
| 170 | BBS2 |  |  | 1643 | 0.057 | 0.3316 | No |
| 171 | PSMD10 |  |  | 1646 | 0.057 | 0.3324 | No |
| 172 | SEPT8 |  |  | 1653 | 0.057 | 0.3326 | No |
| 173 | TOB2 |  |  | 1656 | 0.057 | 0.3335 | No |
| 174 | IQSEC1 |  |  | 1670 | 0.056 | 0.3324 | No |
| 175 | PRCP |  |  | 1675 | 0.056 | 0.3329 | No |
| 176 | NEK1 |  |  | 1687 | 0.056 | 0.3322 | No |
| 177 | IDH1 |  |  | 1691 | 0.055 | 0.3328 | No |
| 178 | TGIF1 |  |  | 1699 | 0.055 | 0.3328 | No |
| 179 | GNG5 |  |  | 1709 | 0.055 | 0.3324 | No |
| 180 | CSGALNACT1 |  |  | 1712 | 0.055 | 0.3332 | No |
| 181 | ZMAT1 |  |  | 1718 | 0.055 | 0.3335 | No |
| 182 | P2RY1 |  |  | 1726 | 0.055 | 0.3335 | No |
| 183 | ITGB8 |  |  | 1765 | 0.053 | 0.3281 | No |
| 184 | SEZ6L |  |  | 1768 | 0.053 | 0.3289 | No |
| 185 | ALDH6A1 |  |  | 1803 | 0.052 | 0.3241 | No |
| 186 | AHCYL1 |  |  | 1811 | 0.052 | 0.3240 | No |
| 187 | WDR41 |  |  | 1823 | 0.052 | 0.3232 | No |
| 188 | ARAP2 |  |  | 1845 | 0.051 | 0.3207 | No |
| 189 | MED13 |  |  | 1847 | 0.051 | 0.3216 | No |
| 190 | SASH1 |  |  | 1848 | 0.051 | 0.3227 | No |
| 191 | MPC1 |  |  | 1881 | 0.050 | 0.3183 | No |
| 192 | C2orf72 |  |  | 1897 | 0.049 | 0.3167 | No |
| 193 | TMEM9B |  |  | 1918 | 0.049 | 0.3143 | No |
| 194 | CRYL1 |  |  | 1922 | 0.049 | 0.3148 | No |
| 195 | DDR1 |  |  | 1941 | 0.048 | 0.3128 | No |
| 196 | LPIN2 |  |  | 1944 | 0.048 | 0.3135 | No |
| 197 | ABI1 |  |  | 1956 | 0.048 | 0.3126 | No |
| 198 | GCSH |  |  | 1971 | 0.048 | 0.3112 | No |
| 199 | LIFR |  |  | 1972 | 0.048 | 0.3122 | No |
| 200 | SLC35F1 |  |  | 1996 | 0.047 | 0.3092 | No |
| 201 | AHCYL2 |  |  | 2013 | 0.047 | 0.3075 | No |
| 202 | FAM120A |  |  | 2020 | 0.046 | 0.3074 | No |
| 203 | NRBP2 |  |  | 2041 | 0.046 | 0.3050 | No |
| 204 | PDLIM3 |  |  | 2070 | 0.045 | 0.3011 | No |
| 205 | PDXK |  |  | 2100 | 0.044 | 0.2971 | No |
| 206 | OAT |  |  | 2106 | 0.044 | 0.2971 | No |
| 207 | PTPRA |  |  | 2113 | 0.043 | 0.2970 | No |
| 208 | IVD |  |  | 2122 | 0.043 | 0.2966 | No |
| 209 | GSTM4 |  |  | 2133 | 0.043 | 0.2958 | No |
| 210 | ACOX1 |  |  | 2140 | 0.043 | 0.2956 | No |
| 211 | PSAT1 |  |  | 2153 | 0.043 | 0.2945 | No |
| 212 | SEL1L3 |  |  | 2171 | 0.042 | 0.2925 | No |
| 213 | NLGN3 |  |  | 2183 | 0.042 | 0.2914 | No |
| 214 | ARHGAP31 |  |  | 2203 | 0.042 | 0.2891 | No |
| 215 | SDC3 |  |  | 2214 | 0.041 | 0.2882 | No |
| 216 | PURB |  |  | 2229 | 0.041 | 0.2867 | No |
| 217 | DPP8 |  |  | 2253 | 0.040 | 0.2836 | No |
| 218 | SPRED1 |  |  | 2272 | 0.040 | 0.2813 | No |
| 219 | PCCB |  |  | 2285 | 0.040 | 0.2801 | No |
| 220 | ALDH5A1 |  |  | 2286 | 0.040 | 0.2809 | No |
| 221 | KCNJ16 |  |  | 2305 | 0.039 | 0.2787 | No |
| 222 | NLGN1 |  |  | 2316 | 0.039 | 0.2778 | No |
| 223 | PHYHIPL |  |  | 2328 | 0.038 | 0.2767 | No |
| 224 | SLC38A3 |  |  | 2359 | 0.038 | 0.2724 | No |
| 225 | SPRY2 |  |  | 2432 | 0.036 | 0.2608 | No |
| 226 | SNX5 |  |  | 2437 | 0.036 | 0.2609 | No |
| 227 | RFX4 |  |  | 2454 | 0.036 | 0.2589 | No |
| 228 | ABLIM1 |  |  | 2473 | 0.035 | 0.2565 | No |
| 229 | PDLIM5 |  |  | 2517 | 0.034 | 0.2499 | No |
| 230 | HSDL2 |  |  | 2546 | 0.034 | 0.2458 | No |
| 231 | NCKAP5 |  |  | 2563 | 0.034 | 0.2438 | No |
| 232 | SNTA1 |  |  | 2571 | 0.033 | 0.2433 | No |
| 233 | TMEM170A |  |  | 2589 | 0.033 | 0.2411 | No |
| 234 | GPRC5B |  |  | 2596 | 0.033 | 0.2407 | No |
| 235 | CAMK2G |  |  | 2599 | 0.033 | 0.2411 | No |
| 236 | RHOJ |  |  | 2605 | 0.033 | 0.2409 | No |
| 237 | AGPAT3 |  |  | 2652 | 0.032 | 0.2337 | No |
| 238 | HMGCS1 |  |  | 2674 | 0.031 | 0.2307 | No |
| 239 | PARP9 |  |  | 2683 | 0.031 | 0.2300 | No |
| 240 | BMPR1B |  |  | 2711 | 0.030 | 0.2260 | No |
| 241 | UGP2 |  |  | 2722 | 0.030 | 0.2250 | No |
| 242 | ZNF521 |  |  | 2732 | 0.030 | 0.2241 | No |
| 243 | IDI1 |  |  | 2749 | 0.030 | 0.2219 | No |
| 244 | SRGAP2B |  |  | 2770 | 0.029 | 0.2191 | No |
| 245 | FGFR3 |  |  | 2779 | 0.029 | 0.2184 | No |
| 246 | ARHGAP12 |  |  | 2794 | 0.029 | 0.2166 | No |
| 247 | ABCD3 |  |  | 2800 | 0.029 | 0.2163 | No |
| 248 | GRIA4 |  |  | 2813 | 0.028 | 0.2148 | No |
| 249 | SPECC1 |  |  | 2875 | 0.027 | 0.2050 | No |
| 250 | LDHB |  |  | 2930 | 0.026 | 0.1962 | No |
| 251 | PPP2R5A |  |  | 2935 | 0.026 | 0.1961 | No |
| 252 | LRP1 |  |  | 2940 | 0.026 | 0.1960 | No |
| 253 | MTMR10 |  |  | 2981 | 0.025 | 0.1896 | No |
| 254 | SIRPA |  |  | 3078 | 0.023 | 0.1737 | No |
| 255 | LRIG1 |  |  | 3101 | 0.023 | 0.1704 | No |
| 256 | PHACTR2 |  |  | 3125 | 0.023 | 0.1669 | No |
| 257 | PTAR1 |  |  | 3126 | 0.023 | 0.1674 | No |
| 258 | F3 |  |  | 3133 | 0.022 | 0.1668 | No |
| 259 | USP46 |  |  | 3142 | 0.022 | 0.1659 | No |
| 260 | IL6ST |  |  | 3218 | 0.021 | 0.1535 | No |
| 261 | ROBO3 |  |  | 3248 | 0.020 | 0.1490 | No |
| 262 | MEGF10 |  |  | 3315 | 0.018 | 0.1380 | No |
| 263 | CTNNB1 |  |  | 3329 | 0.017 | 0.1361 | No |
| 264 | ABCA3 |  |  | 3335 | 0.017 | 0.1357 | No |
| 265 | S100A13 |  |  | 3355 | 0.017 | 0.1327 | No |
| 266 | MLLT6 |  |  | 3372 | 0.016 | 0.1303 | No |
| 267 | IGDCC4 |  |  | 3402 | 0.016 | 0.1257 | No |
| 268 | CAMK2D |  |  | 3403 | 0.016 | 0.1260 | No |
| 269 | MYO6 |  |  | 3404 | 0.015 | 0.1264 | No |
| 270 | NLGN4X |  |  | 3412 | 0.015 | 0.1255 | No |
| 271 | DPM3 |  |  | 3486 | 0.013 | 0.1132 | No |
| 272 | LGALS1 |  |  | 3527 | 0.011 | 0.1066 | No |
| 273 | FMNL2 |  |  | 3528 | 0.011 | 0.1068 | No |
| 274 | TJP1 |  |  | 3531 | 0.011 | 0.1067 | No |
| 275 | GAS1 |  |  | 3553 | 0.011 | 0.1034 | No |
| 276 | LDLRAD3 |  |  | 3586 | 0.009 | 0.0981 | No |
| 277 | ITM2C |  |  | 3587 | 0.009 | 0.0983 | No |
| 278 | ARHGEF26 |  |  | 3596 | 0.009 | 0.0971 | No |
| 279 | TMEM132B |  |  | 3600 | 0.009 | 0.0967 | No |
| 280 | SNAP23 |  |  | 3616 | 0.008 | 0.0944 | No |
| 281 | COMT |  |  | 3621 | 0.008 | 0.0938 | No |
| 282 | SWAP70 |  |  | 3664 | 0.006 | 0.0868 | No |
| 283 | TMEM106B |  |  | 3704 | 0.005 | 0.0802 | No |
| 284 | CNDP2 |  |  | 3714 | 0.005 | 0.0787 | No |
| 285 | TNFRSF19 |  |  | 3725 | 0.004 | 0.0771 | No |
| 286 | ITPK1 |  |  | 3750 | 0.003 | 0.0731 | No |
| 287 | LGALS3BP |  |  | 3757 | 0.003 | 0.0721 | No |
| 288 | LSAMP |  |  | 3779 | 0.001 | 0.0685 | No |
| 289 | VEGFA |  |  | 3787 | 0.001 | 0.0673 | No |
| 290 | IFT88 |  |  | 3800 | 0.001 | 0.0653 | No |
| 291 | CD99 |  |  | 3814 | 0.000 | 0.0631 | No |
| 292 | EFHD2 |  |  | 3841 | -0.001 | 0.0586 | No |
| 293 | OLIG1 |  |  | 3854 | -0.002 | 0.0566 | No |
| 294 | SOX8 |  |  | 3882 | -0.003 | 0.0521 | No |
| 295 | DUSP6 |  |  | 3911 | -0.004 | 0.0473 | No |
| 296 | STAT3 |  |  | 3937 | -0.006 | 0.0432 | No |
| 297 | NOTCH2 |  |  | 3944 | -0.006 | 0.0423 | No |
| 298 | TLK1 |  |  | 3945 | -0.006 | 0.0424 | No |
| 299 | PARP14 |  |  | 3950 | -0.007 | 0.0419 | No |
| 300 | NTRK3 |  |  | 3955 | -0.007 | 0.0413 | No |
| 301 | SLC35A5 |  |  | 3972 | -0.008 | 0.0388 | No |
| 302 | JUN |  |  | 3979 | -0.008 | 0.0379 | No |
| 303 | MAP3K1 |  |  | 4018 | -0.010 | 0.0316 | No |
| 304 | SPATA6 |  |  | 4039 | -0.012 | 0.0284 | No |
| 305 | PDE4B |  |  | 4063 | -0.013 | 0.0248 | No |
| 306 | TAPBP |  |  | 4109 | -0.016 | 0.0174 | No |
| 307 | DBI |  |  | 4122 | -0.017 | 0.0157 | No |
| 308 | GJA1 |  |  | 4123 | -0.017 | 0.0160 | No |
| 309 | GABBR1 |  |  | 4170 | -0.020 | 0.0086 | No |
| 310 | ZNF236 |  |  | 4171 | -0.020 | 0.0090 | No |
| 311 | MEIS1 |  |  | 4218 | -0.023 | 0.0016 | No |
| 312 | ZMYM2 |  |  | 4258 | -0.026 | -0.0046 | No |
| 313 | LPP |  |  | 4265 | -0.026 | -0.0050 | No |
| 314 | LARS |  |  | 4285 | -0.028 | -0.0077 | No |
| 315 | DAG1 |  |  | 4317 | -0.030 | -0.0124 | No |
| 316 | NOTCH1 |  |  | 4321 | -0.031 | -0.0123 | No |
| 317 | ID4 |  |  | 4329 | -0.031 | -0.0128 | No |
| 318 | PSENEN |  |  | 4339 | -0.031 | -0.0137 | No |
| 319 | NTM |  |  | 4381 | -0.034 | -0.0200 | No |
| 320 | TNK2 |  |  | 4382 | -0.034 | -0.0193 | No |
| 321 | PNISR |  |  | 4388 | -0.035 | -0.0194 | No |
| 322 | ZNF621 |  |  | 4428 | -0.038 | -0.0253 | No |
| 323 | LRP1B |  |  | 4460 | -0.041 | -0.0297 | No |
| 324 | JAG1 |  |  | 4496 | -0.044 | -0.0348 | No |
| 325 | ALCAM |  |  | 4499 | -0.045 | -0.0342 | No |
| 326 | MAML2 |  |  | 4512 | -0.045 | -0.0353 | No |
| 327 | PTCH1 |  |  | 4529 | -0.046 | -0.0371 | No |
| 328 | ILDR2 |  |  | 4550 | -0.048 | -0.0395 | No |
| 329 | IFNAR1 |  |  | 4576 | -0.049 | -0.0428 | No |
| 330 | SYT11 |  |  | 4599 | -0.051 | -0.0454 | No |
| 331 | HIBCH |  |  | 4615 | -0.052 | -0.0469 | No |
| 332 | ATP9B |  |  | 4626 | -0.053 | -0.0475 | No |
| 333 | FKBP10 |  |  | 4638 | -0.054 | -0.0483 | No |
| 334 | CCDC80 |  |  | 4657 | -0.056 | -0.0502 | No |
| 335 | BCHE |  |  | 4672 | -0.057 | -0.0514 | No |
| 336 | RNF180 |  |  | 4674 | -0.057 | -0.0503 | No |
| 337 | FJX1 |  |  | 4678 | -0.058 | -0.0496 | No |
| 338 | LRRC4C |  |  | 4682 | -0.058 | -0.0489 | No |
| 339 | PDGFRA |  |  | 4697 | -0.059 | -0.0501 | No |
| 340 | SCD |  |  | 4717 | -0.061 | -0.0520 | No |
| 341 | BMP7 |  |  | 4727 | -0.062 | -0.0523 | No |
| 342 | MEST |  |  | 4739 | -0.063 | -0.0528 | No |
| 343 | IFI6 |  |  | 4742 | -0.063 | -0.0518 | No |
| 344 | PLOD2 |  |  | 4764 | -0.065 | -0.0541 | No |
| 345 | STK17A |  |  | 4774 | -0.066 | -0.0542 | No |
| 346 | PRNP |  |  | 4778 | -0.066 | -0.0533 | No |
| 347 | S100A16 |  |  | 4787 | -0.067 | -0.0533 | No |
| 348 | MRPS5 |  |  | 4848 | -0.072 | -0.0621 | No |
| 349 | ENO1 |  |  | 4868 | -0.073 | -0.0638 | No |
| 350 | MTRNR2L8 |  |  | 4898 | -0.075 | -0.0672 | No |
| 351 | CLSTN1 |  |  | 4906 | -0.075 | -0.0668 | No |
| 352 | PKM |  |  | 4921 | -0.077 | -0.0676 | No |
| 353 | FAM3C |  |  | 4929 | -0.077 | -0.0671 | No |
| 354 | PCDHGC3 |  |  | 4939 | -0.078 | -0.0670 | No |
| 355 | PTPRG |  |  | 4952 | -0.079 | -0.0674 | No |
| 356 | SCRG1 |  |  | 4970 | -0.080 | -0.0686 | No |
| 357 | PCDH9 |  |  | 4972 | -0.080 | -0.0671 | No |
| 358 | CPE |  |  | 5002 | -0.083 | -0.0703 | No |
| 359 | ITM2B |  |  | 5050 | -0.087 | -0.0766 | No |
| 360 | HSD17B12 |  |  | 5056 | -0.087 | -0.0756 | No |
| 361 | COL6A1 |  |  | 5059 | -0.088 | -0.0741 | No |
| 362 | TMED10 |  |  | 5078 | -0.089 | -0.0753 | No |
| 363 | SERPINH1 |  |  | 5124 | -0.094 | -0.0810 | No |
| 364 | ZFYVE16 |  |  | 5141 | -0.096 | -0.0818 | No |
| 365 | SCCPDH |  |  | 5153 | -0.097 | -0.0816 | No |
| 366 | AASS |  |  | 5154 | -0.097 | -0.0795 | No |
| 367 | JAM2 |  |  | 5167 | -0.099 | -0.0795 | No |
| 368 | GPC6 |  |  | 5195 | -0.101 | -0.0820 | No |
| 369 | FGFBP3 |  |  | 5233 | -0.104 | -0.0862 | No |
| 370 | CLDND1 |  |  | 5238 | -0.104 | -0.0847 | No |
| 371 | DST |  |  | 5242 | -0.105 | -0.0830 | No |
| 372 | PON2 |  |  | 5246 | -0.105 | -0.0813 | No |
| 373 | ABHD2 |  |  | 5255 | -0.107 | -0.0804 | No |
| 374 | PTN |  |  | 5261 | -0.107 | -0.0790 | No |
| 375 | DPP6 |  |  | 5279 | -0.108 | -0.0796 | No |
| 376 | DAD1 |  |  | 5287 | -0.109 | -0.0785 | No |
| 377 | ITGAV |  |  | 5309 | -0.110 | -0.0798 | No |
| 378 | CADM4 |  |  | 5313 | -0.110 | -0.0780 | No |
| 379 | EGFR |  |  | 5316 | -0.110 | -0.0760 | No |
| 380 | TTC14 |  |  | 5322 | -0.111 | -0.0745 | No |
| 381 | SPPL2A |  |  | 5336 | -0.112 | -0.0744 | No |
| 382 | TNC |  |  | 5359 | -0.114 | -0.0758 | No |
| 383 | MCFD2 |  |  | 5403 | -0.118 | -0.0806 | No |
| 384 | SFXN5 |  |  | 5408 | -0.120 | -0.0788 | No |
| 385 | UFL1 |  |  | 5413 | -0.120 | -0.0770 | No |
| 386 | PSME2 |  |  | 5418 | -0.121 | -0.0751 | No |
| 387 | CD81 |  |  | 5427 | -0.122 | -0.0739 | No |
| 388 | CERS6 |  |  | 5431 | -0.122 | -0.0719 | No |
| 389 | PNPT1 |  |  | 5531 | -0.135 | -0.0860 | No |
| 390 | FADS2 |  |  | 5544 | -0.135 | -0.0852 | No |
| 391 | PLEKHH2 |  |  | 5547 | -0.136 | -0.0827 | No |
| 392 | SMC5 |  |  | 5557 | -0.136 | -0.0813 | No |
| 393 | GPM6A |  |  | 5563 | -0.137 | -0.0793 | No |
| 394 | ITGA6 |  |  | 5574 | -0.139 | -0.0781 | No |
| 395 | SLC7A11 |  |  | 5582 | -0.141 | -0.0763 | No |
| 396 | LIMA1 |  |  | 5593 | -0.144 | -0.0750 | No |
| 397 | SYPL1 |  |  | 5607 | -0.145 | -0.0742 | No |
| 398 | SLC25A5 |  |  | 5628 | -0.148 | -0.0745 | No |
| 399 | EPB41L2 |  |  | 5665 | -0.154 | -0.0774 | No |
| 400 | MFGE8 |  |  | 5668 | -0.154 | -0.0745 | No |
| 401 | CDCA7L |  |  | 5720 | -0.164 | -0.0798 | No |
| 402 | MMP16 |  |  | 5734 | -0.168 | -0.0785 | No |
| 403 | CANX |  |  | 5755 | -0.173 | -0.0782 | No |
| 404 | WSB1 |  |  | 5773 | -0.178 | -0.0774 | No |
| 405 | IGFBP2 |  |  | 5777 | -0.179 | -0.0741 | No |
| 406 | MALAT1 |  |  | 5783 | -0.179 | -0.0712 | No |
| 407 | NOVA1 |  |  | 5789 | -0.181 | -0.0683 | No |
| 408 | SLC16A1 |  |  | 5794 | -0.182 | -0.0651 | No |
| 409 | PTPRZ1 |  |  | 5811 | -0.186 | -0.0639 | No |
| 410 | B2M |  |  | 5823 | -0.188 | -0.0618 | No |
| 411 | AXL |  |  | 5826 | -0.189 | -0.0582 | No |
| 412 | TMBIM6 |  |  | 5855 | -0.196 | -0.0589 | No |
| 413 | MSMO1 |  |  | 5866 | -0.198 | -0.0564 | No |
| 414 | FAT1 |  |  | 5898 | -0.206 | -0.0574 | No |
| 415 | SERPINE2 |  |  | 5913 | -0.210 | -0.0554 | No |
| 416 | TACC1 |  |  | 5938 | -0.218 | -0.0549 | No |
| 417 | RCN1 |  |  | 5945 | -0.219 | -0.0513 | No |
| 418 | MAGI1 |  |  | 5985 | -0.233 | -0.0531 | No |
| 419 | LAPTM4B |  |  | 5994 | -0.236 | -0.0495 | No |
| 420 | SLC2A1 |  |  | 5995 | -0.237 | -0.0445 | No |
| 421 | MLEC |  |  | 5997 | -0.237 | -0.0396 | No |
| 422 | HNRNPA2B1 |  |  | 6029 | -0.251 | -0.0397 | No |
| 423 | PPIB |  |  | 6033 | -0.256 | -0.0348 | No |
| 424 | SSR3 |  |  | 6046 | -0.267 | -0.0312 | No |
| 425 | GOLIM4 |  |  | 6059 | -0.273 | -0.0275 | No |
| 426 | DSEL |  |  | 6121 | -0.316 | -0.0313 | No |
| 427 | PHGDH |  |  | 6126 | -0.318 | -0.0253 | No |
| 428 | CD9 |  |  | 6176 | -0.378 | -0.0257 | No |
| 429 | TIMP1 |  |  | 6191 | -0.409 | -0.0195 | No |
| 430 | BCAN |  |  | 6211 | -0.481 | -0.0126 | No |
| 431 | CD63 |  |  | 6220 | -0.496 | -0.0035 | No |
| 432 | HSP90B1 |  |  | 6225 | -0.509 | 0.0065 | No |
Table: GSEA details [plain text format]

  

Fig 2: RICHARDS\_NOWAKOWSKI\_SCIENCE\_2017\_ASTROCYTE\_UPREG: Random ES distribution      
 Gene set null distribution of ES for **RICHARDS\_NOWAKOWSKI\_SCIENCE\_2017\_ASTROCYTE\_UPREG**
